# The COVID-19 experience among international migrant workers in the Republic of Korea: knowledge and awareness of treatment and immigration policies

**DOI:** 10.1186/s12889-024-20790-5

**Published:** 2024-11-27

**Authors:** Jayoung Park, Jongho Heo, Woong-Han Kim, Sugy Choi

**Affiliations:** 1 Department of Human Systems Medicine, Seoul National University College of Medicine, Seoul, Korea; 2 Program in Global Surgery and Implementation Science, JW LEE Center for Global Medicine, Seoul National University College of Medicine, Seoul, the Republic of Korea; 3 Department of Thoracic and Cardiovascular Surgery, Seoul National University College of Medicine, Seoul, Republic of Korea; 4 Department of Thoracic and Cardiovascular Surgery, Seoul National University Children’s Hospital, Seoul, Republic of Korea; 5 National Assembly Futures Institute, Seoul, Republic of Korea; 6 Department of Population Health, NYU Grossman School of Medicine, New York, NY USA

**Keywords:** International migrant workers, Undocumented migrant workers, COVID-19 pandemic, Health insurance, Health policy

## Abstract

**Introduction:**

The COVID-19 pandemic has exposed various health risks and inequities experienced by international migrant workers. The number of migrant workers in the Republic of Korea (ROK) is rapidly growing and is expected to continue growing. Health related research on migrant workers in ROK is limited, especially among undocumented migrant workers who were more vulnerable to the pandemic. This study aims to examine the experiences of migrant workers and their knowledge and awareness of treatment and immigration policies during the pandemic.

**Methods:**

We used data from the International Migrant Workers’ COVID-19 Health Literacy and Access to Medical Care project, a cross-sectional survey conducted with international migrant workers residing in ROK in 2021 (*n* = 537). Descriptive statistics and multivariable regression models were employed to understand different demographic, occupational, and immigration factors affecting migrant workers’ knowledge and awareness of treatment and immigration policies.

**Results:**

Undocumented migrant workers had a longer length of residence in ROK and earned less compared to workers with work visa status. None of the undocumented migrant workers had access to health insurance since they were ineligible to enroll in the national health insurance scheme. In the early days of the pandemic, most undocumented migrant workers experienced a decrease in their average income. After adjusting for demographic differences and language proficiency, undocumented migrant workers (AOR: 0.41, 95% CI: 0.21, 0.78) were less likely to be aware of the policy allowing foreigners, including undocumented individuals, to access COVID-19 testing and treatment without the risk of deportation. Workers with a longer length of residence (AOR: 1.29, 95% CI: 1.09, 1.53) were more likely to be aware of this policy.

**Conclusion:**

Undocumented migrant workers were often less informed about COVID-19 policies. While most of the survey respondents were knowledgeable about governmental policies regarding COVID-19 treatment and immigration, our results reveal multiple occupational and health insurance vulnerabilities of undocumented migrant workers living in ROK. More attention is needed to understand healthcare service barriers and how to provide adequate resources for this vulnerable population.

## Introduction

During a pandemic, public health measures can neglect the health needs of international migrant workers [1, 2]. The COVID-19 pandemic has served as a critical reminder to the international community of the urgency to address the safety, health, and well-being of migrant workers [3, 4]. The oversight of migrant workers’ health needs has led to an increased risk of COVID-19 transmissions and has placed additional strain on healthcare systems [5, 6].

Recognizing the significance of this issue, the International Labour Organization (ILO) and the World Health Organization (WHO) have emphasized the importance of addressing the health of international migrant workers during the COVID-19 pandemic. The ILO has called for the protection of the health and safety of migrant workers, especially those in high-risk occupations, through measures such as providing adequate protective equipment, hygiene measures, and healthcare services [7]. The ILO brief highlights the importance of policies that safeguard the labor rights and social protections of migrant workers, such as ensuring sick leave, wage protections, and safe working conditions [8]. Similarly, the WHO recognizes the health risks faced by migrant workers and the negative consequences of neglecting their health for both workers and the wider community [9]. The WHO has provided guidance on measures to protect the health of migrant workers during the pandemic, such as ensuring access to healthcare and COVID-19 testing, providing adequate living and working conditions, and offering social protections for those who have lost their jobs or income due to the pandemic.

Despite these global efforts, countries like the Republic of Korea (ROK), historically characterized by a homogeneous population, face challenges in crafting migration and health policies that effectively address the specific needs of international migrant workers during the COVID-19 pandemic. In recent years, ROK has experienced domestic transitions, including a declining population and a job skills mismatch that has made it difficult for the domestic population to meet the labor demands of small and medium-sized enterprises [10]. Additionally, migrant workers often experience power imbalance in employer-employee relationship, exposing them to exploitation [11–14]. Undocumented migrant workers often lack access to basic labor rights [15, 16] and may be hesitant to report workplace abuses or safety violations for fear of deportation, further exacerbating their vulnerability [17]. Despite these obstacles, ROK is in the early stages of embracing multiculturalism, with limited incentives or political will to enhance conditions for both documented and undocumented migrant workers.

The COVID-19 pandemic has underscored the challenges faced by international migrant workers, particularly those who are undocumented. These individuals often hesitate to seek medical care due to fear of being reported to authorities or due to concerns about their immigration status [18]. Such reluctance can lead to delayed or inadequate care, worsening existing health conditions and increasing their vulnerability to COVID-19 [19]. In line with these concerns, a recent qualitative study on the healthcare experiences of migrant workers in ROK during the pandemic revealed that these workers faced significant difficulties in accessing and using healthcare services due to linguistic barriers and a lack of adequate support systems [20]. Furthermore, migrant workers often have difficulties with language [21] so they are unfamiliar when new COVID-19 rules and measures imposed by the host-country government [22, 23]. Despite the critical insights provided by previous studies, there has been limited research specifically examining the vulnerabilities of migrant workers based on their resident status during the COVID-19 pandemic. Therefore, this study aimed to explore the awareness and knowledge of treatment and immigration policies of international migrant workers living in ROK during the COVID-19 pandemic.

## Methods

### Study design and participants

We used the International Migrant Worker’s COVID-19 Health Literacy and Access to Medical Care dataset, a cross-sectional survey collected in 2021 in ROK. To achieve the pre-determined sample size of 500, a total of 550 individuals were contacted, accounting for a potential non-response rate of 10%. The sample was selected according to specific quotas based on documented status (1:1 ratio of documented to undocumented), gender (1:1 ratio of female to male), and employment status (3:7 ratio of unemployed to employed). Undocumented individuals were intentionally oversampled to ensure reliable estimates. In total, 528 international migrant workers were recruited, including documented migrant workers with E-9 visa status under ROK’s Employment Permit System (EPS) and undocumented foreign residents without visa status. Participants were recruited from Seoul and Gyeonggi province through quota sampling methods [24, 25]. Recruitment venues included migrant worker centers, religious facilities, and Korean language schools.

### Data collection

A self-administered survey was developed using four domains: (1) employment status and condition, (2) health factors, (3) understanding of COVID-19, and (4) socio-demographic information. The original English text was translated to Korean, Nepali, Russian, Vietnamese, Uzbek, Thai, and Tagalog. It was then back-translated from the translated languages to English by another translator. The study team compared the back-translated survey to the original questionnaire. The questionnaires were edited collaboratively by translators and the study team until a consensus was reached when inconsistencies were identified.

Between February 16, 2021, and March 26, 2021, during the third wave of the COVID-19 pandemic in ROK, a trained study team collected survey data after obtaining written consent from participants. Participants were assured that the study was completely anonymous and that no personal identifiers would be collected. Consent forms were provided, clearly outlining the voluntary participation and the right to withdraw from the study at any time without any consequences. In appreciation for their participation, participants received approximately US $10.

### Measures

#### Perception and knowledge of COVID-19 testing and treatment policies

Participants were surveyed regarding their awareness of specific COVID-19 testing and treatment policies. The questions assessed whether they knew the following policies:

1. Foreigners (including non-registered individuals) may receive COVID-19 testing and treatment without forced departure.
2. If it is difficult to return to your home country due to COVID-19, you can stay in ROK for 50 days longer even if the period of work is over, or you can stay until it is possible to leave ROK.

#### Residence status

Participants were asked about their residence status with the question: “When you first entered the country, what was your residence qualification?” The possible responses were either “work visa” or “undocumented.”

#### Occupational factors

Occupational characteristics included the following factors: current employment status, part-time work status, monthly income, employment status, and employee experience since COVID-19 (including work experience, loss of main source of income, decrease in average income, and reduced average monthly income).

#### Healthcare factors

Participants were asked about their access to health insurance, specifying the type of health insurance and reasons for being uninsured.

#### Socio-demographic factors

Sociodemographic factors included their nationality, gender, age, education, marital status, living arrangements with a spouse/partner in ROK, length of residency, and proficiency in Korean.

### Statistical analysis

Sociodemographic and occupational characteristics, employee experience, access to health insurance, knowledge and awareness of COVID-19 treatment and immigration policies, and COVID-19 testing uptake of international workers living in ROK by their status of residence (work visa or undocumented) were assessed using an independent sample t-test, chi-square, or one-way Analysis of Variance (ANOVA). The level of statistical significance was set at *p* < 0.01.

Multivariable regression analyses were performed to assess the association of the chosen independent variables with understanding COVID-19 policies among international migrant workers in ROK. Model 1 assessed how occupational characteristics and sociodemographic factors affect understanding of a policy regarding foreigners having the option of getting tested and treated for COVID-19 without having to leave. In Model 2, the same factors were examined for their association with the knowledge that foreigners can stay in ROK for an additional 50 days after their visa expires if they cannot return home because of COVID-19. Model 3 examined the impact of factors on COVID-19 testing among those with a work visa (*n* = 278). *P* values less than 0.05 were considered statistically significant. The results are presented as adjusted odds ratios (OR) with 95% confidence intervals (95% CI). All statistical analyses were done using the StataCorp. 2021. *Stata Statistical Software: Release 17*. College Station, TX: StataCorp LLC.

## Results

A total of 528 international migrant workers participated in the study and 278 (52.7%) of them had a work visa and 250 (47.3%) were undocumented during the COVID-19 pandemic.

**Table 1:** Demographic and occupational characteristics and workplace experiences of international migrant workers living in the ROK by current status of residence

	Work visa *n* = 278	Undocumented *n* = 250	*p*-value
**Length of residence (year)**,** mean (sd)**	3.8 (2.07)	5.6 (2.45)	< 0.001
**Nationality**			0.093
Central Asian	16.9	12.8	
South Asian	16.6	16.4	
Southeast Asian	65.1	66.0	
Other	1.4	4.8	
**Sex**,** %**			0.226
Male	52.9	47.6	
Female	47.1	52.4	
**Age**,** %**			< 0.001
20–30	28.4	18.4	
31–40	51.1	70.8	
41+	20.5	10.8	
**Educational attainment**,** %**			0.068
≤ 12 years	66.9	72.4	
> 12 years	32.4	25.2	
Refuse to answer	0.7	2.4	
**Marital status**,** %**			0.090
Currently married	37.8	48.4	
Never married	55.8	46.0	
Not currently married	2.5	2.8	
Refuse to answer	4.0	2.8	
**If married**,** spouse/partner is currently living in Korea**,** %**	11.4	14.1	0.557
**Currently unemployed**,** %**	26.6	32.8	0.120
**Working part-time**, %*	0.0	16.1	< 0.001
**Monthly income (KRW)**, %*			< 0.001
Less than 1,000,000	0.0	9.5	
1,000,000–1,500,000	15.7	50.0	
1,500,000–2,000,000	63.4	30.4	
More than 2,000,000	21.1	10.1	
**Korean proficiency**			0.678
High	28.4	26.8	
Low	71.6	73.2	
**Workplace experience**,** %**			
Laid off/fired	0.0	7.6	< 0.001
Reduced payroll	19.4	25.6	0.089
Flexible hours	28.8	34.4	0.165
Unpaid leave	8.6	15.2	< 0.001
**Lost main source of income**,** %**	25.5	38.4	0.002
**Experienced a decrease in average income**,** %**	59.0	72.4	< 0.001
**Reduced average monthly income**, KRW** (sd)	885,182 (730,053)	661,436 (473,021)	< 0.001

Note: *Among working individuals (*n* = 372); **if experienced a decrease in average income

Table 1 depicts the socio-demographic and occupational characteristics of international migrant workers living in ROK during the COVID-19 pandemic. Undocumented migrant workers had a longer length of residence in ROK compared to migrant workers with work visa status (M = 5.6 vs. 3.8 years). Among undocumented migrant workers, 16.1% worked part-time, while none of the migrant workers with visas were part-time. Approximately 20% of migrant workers with a work visa earned more than 2 million Korean Republic Won (KRW) per month, whereas only 10% of undocumented migrant workers earned more than 2 million KRW per month. Amid the COVID-19 pandemic, 7.6% of undocumented migrant workers were laid off or fired from their workplaces, whereas no migrant workers with visas faced the same situation. Additionally, 15.2% of undocumented migrant workers and 8.6% of migrant workers with visas experienced unpaid leaves. Both undocumented migrant workers and migrant workers with visas suffered losses in their main source of income, with over a quarter of each group affected. More than half of these workers reported a decrease in their average income.

**Table 2:** Access to health insurance among international migrant workers during COVID-19

	Residence status		
	Work visa (*n* = 278)	Undocumented (*n* = 250)	*p*-value
**Uninsured**,** %**	8.6	100.0	< 0.001
**Type of health insurance**, %*			
Employee National Health Insurance (NHI),	76.4	-	
Self-employed NHI	20.5	-	
Mutual aid	3.2	-	
**Reasons for being uninsured**, %**			< 0.001
Not affordable	50.0	0.0	
Signing up too difficult/confusing	4.2	0.0	
Do not need (able to get care without insurance)	45.8	0.0	
Not eligible	0.0	100.0	

Note: *if insured *n* = 254, ** if uninsured *n* = 274

Table 2 shows access to health insurance among international migrant workers during the COVID-19 pandemic categorized by their current status of residence. None of the undocumented migrant workers had health insurance, as they were ineligible (100%) for it, resulting in being uninsured.

**Table 3:** Knowledge and awareness of COVID-19 treatment and immigration policies and COVID-19 testing uptake

	Residence status		
	Work visa (*n* = 278)	Undocumented (*n* = 250)	*p*-value
Aware that foreigners (including undocumented) can also be tested and treated for COVID-19 without being forced to leave the country, %	92.5	87.2	0.045
Aware that when the visa expires and it is difficult to return home due to the COVID-19, foreigners can stay in Korea for an additional 50 days, or until it is possible to leave, %	78.8	81.2	0.488
COVID-19 testing uptake, %	31.3	0	< 0.001

Table 3 displays descriptive results regarding knowledge and awareness of COVID-19 treatment and immigration policies, as well as COVID-19 testing uptake. Workers with a visa (92.5%) demonstrated higher awareness than undocumented migrant workers (87.2%) regarding the fact that foreigners (including undocumented) can also be tested and treated for COVID-19 without being forced to leave the country. The COVID-19 testing uptake results indicate that 31.3% of those with work visas have undergone COVID-19 testing, while undocumented migrant workers have not received any testing.

**Table 4:** Factors associated with understanding COVID-19 policies among international migrant workers in ROK

	Full sample AOR (95% CI)		Work visa AOR (95% CI)
	Aware that foreigners (including undocumented) can also be tested and treated for COVID-19 without being forced to leave the country. AOR (95% CI)	Aware that when the visa expires and it is difficult to return home due to the COVID-19, foreigners can stay in Korea for an additional 50 days, or until it is possible to leave.	Tested for COVID-19
**Undocumented**	0.41** (0.21, 0.78)	1.16 (0.71, 1.89)	N/A
**Length of residence (years)**	1.29** (1.09, 1.53)	1.21** (1.07, 1.37)	0.88 (0.76, 1.03)
**Male**	0.86 (0.48, 1.57)	0.68 (0.43, 1.06)	0.72 (0.41, 1.27)
**Age group**			
20–30	Ref	Ref	Ref
31–40	1.14 (0.59, 2.19)	1.88* (1.16, 3.07)	0.40** (0.22, 0.73)
41+	4.33 (0.94 19.90)	5.02** (1.98, 12.8)	0.16*** (0.06, 0.41)
**Currently employed**	0.71 (0.38, 1.33)	1.14 (0.69, 1.90)	0.66 (0.33, 1.31)
**Korean proficiency level**			
High	0.92 (0.46, 1.82)	0.78 (0.47, 1.29)	0.93 (0.49, 1.74)
Observations	528	528	278
R-squared	0.0724	0.0728	0.0974

*p ≤ 0.05, **p ≤ 0.01, ***p ≤ 0.001

Ref, reference category

Table 4 displays the multivariable regression analysis results. After controlling for demographic differences and language proficiency, undocumented migrant workers (AOR: 0.41, 95% CI: 0.21, 0.78) were less likely to be aware that foreigners (including undocumented individuals) can also be tested and treated for COVID-19 without being forced to leave the country. Workers with a longer length of residence (AOR: 1.29, 95% CI: 1.09, 1.53) were more likely to be aware of aforementioned policy.

## Discussion

Our study examined the COVID-19 experiences of international migrant workers living in ROK, providing insights into their socio-demographic status, occupational characteristics, work experiences, knowledge of COVID-19 policies, and access to health care services during the pandemic. We found that the pandemic had a negative influence on their work experience, including job losses, unpaid leaves, and income reductions, regardless of their visa status. Notably, none of undocumented migrant workers had health insurance as they were ineligible to enroll in the national health insurance scheme. Most of the interviewees knew about the governmental policies on COVID-19 treatment and immigration; however, undocumented migrant workers were less informed of the policies compared to workers with visas.

Since undocumented international migrant workers were less likely to be informed about the COVID-19 policies than those with visas, this indicates that public health communication can be improved. Previous research has suggested several strategies for enhancing communication and information access for this vulnerable group, including expanding communication channels, providing multilingual resources, simplifying information, and collaborating with community organizations [26]. Despite the Korean government’s efforts to distribute COVID-19 policies in multiple languages, there is still a possibility that the information provided may not be easily understood or accessible for undocumented migrant workers who often live in isolated living and working conditions [27, 28]. This could hinder their access to important information and resources related to COVID-19, creating a significant barrier. To ensure that all migrant workers, especially the most vulnerable, have access to clear, concise, and easily accessible information, the government must make additional efforts to improve communication and provide necessary resources.

Similar to previous studies, our research highlights the disproportionate economic impact of the pandemic on undocumented migrant workers, exposing them to even greater financial insecurity, especially when they become ill during the pandemic [29, 30]. Despite the financial insecurity, undocumented migrant workers were excluded from receiving governmental benefits during COVID-19. Undocumented migrants were eligible for testing and treatment services, but not financial assistance such as emergency relief grants [31]. In ROK, starting from September 2021, the government announced that all Korean nationals would receive emergency relief grant of 250,000 won ($215) per person in a bid to ease economic hardship from the COVID-19 pandemic. This monetary support was only offered to permanent residents (F-5 visa holders) and marriage immigrants (F-6 visa holders) [32] leaving the majority of non-nationals without support [31].

As is the case in many countries, undocumented international migrant workers in ROK are not eligible to enroll in the National Health Insurance system [33]. While private insurance options are available, our study revealed that none of the participants had insurance coverage. Consequently, out-of-pocket payments are high, deterring them from seeking treatment and exacerbating their health conditions [34]. While some alternative options for minimal health care services exist for undocumented migrant workers, such as free medical care campaigns organized by nonprofit organizations like the Raphael Clinic [35] and Seoul Migrant Workers Center [36], these initiatives have been curtailed or suspended due to COVID-19 restrictions [37], preventing migrant workers from obtaining healthcare.

One of the ways that ROK can improve their universal health care coverage is to initiate access to health services for undocumented international migrant workers. This could involve free or low-cost services from non-profit providers. For example, Thailand’s Ministry of Public Health has implemented the Migration-Fund (M-Fund) Health Insurance Program, offering a voluntary, low-cost, and non-profit health insurance scheme for migrant workers [38]. This program covers all migrant workers, regardless of their documentation status.

The results should be viewed within the context of some limitations. As a cross-sectional survey, it restricts our ability to establish causal relationships. While the dataset comprises 528 survey respondents, which is a substantial number of migrant individuals, it is important to consider the geographical limitations inherent in the data collection process. Specifically, the data were gathered exclusively from Seoul and Gyeonggi-do, implying that other areas were not included in the study. Furthermore, several undocumented international migrant workers declined to participate, likely due to fears related to their immigration status. This led to a selection bias, as those who participated may not fully represent the broader undocumented migrant worker population. Despite these limitations, this research is currently the only quantitative study involving both documented and undocumented migrant workers in the ROK, with a robust sample size, offering valuable insights into this population’s experiences.

Future research should focus on better understanding the COVID-19 incidence rate and testing behaviors among migrants. As mentioned earlier, this group faces unique risks and barriers to healthcare access, necessitating detailed studies to assess infection rates and specific risk factors. Employing mixed methods approaches and triangulating data from multiple sources could provide a more comprehensive understanding of the issues faced by undocumented international migrant workers and inform more effective and equitable health policies and interventions. Future studies should also aim to broaden recruitment locations to improve the generalizability of the findings. Additionally, exploring the intricate relationship between migrant employment and health during a pandemic and identifying effective communication strategies for underserved populations during national emergencies is crucial. Furthermore, we recommend that future research focuses on collecting and analyzing data on testing behaviors to address this important aspect comprehensively.

## Conclusion

In conclusion, this study highlights the health disparities faced by international migrant workers in ROK during the COVID-19 pandemic. We found that while many migrant workers in ROK were informed about the nation’s COVID-19 policies, a significant disparity existed, particularly with undocumented migrant workers who often lack the same level of awareness. The study underscores the economic hardships experienced by undocumented migrant workers due to their exclusion from governmental benefits, suggesting the importance of more inclusive relief measures. Additionally, there is a need to develop improved communication strategies and multilingual resources to ensure that all migrant workers, irrespective of their documentation status, receive important information.

## Data Availability

The datasets used and/or analyzed during the current study available from the corresponding author on reasonable request.

## Abbreviations

AOR: Adjusted odds ratio
CI: Confidence interval
EPS: Employment Permit System
ILO: International Labour Organization
KRW: Korean Republic Won
M-Fund: Migration-Fund
ROK: Republic of Korea
WHO: World Health Organization
